# Impact of nutritional index on long-term outcomes of elderly patients with coronary artery disease: sub-analysis of the SHINANO 5 year registry

**DOI:** 10.1007/s00380-020-01659-0

**Published:** 2020-06-30

**Authors:** Shusaku Maruyama, Souichiro Ebisawa, Takashi Miura, Hisanori Yui, Daisuke Kashiwagi, Ayumu Nagae, Takahiro Sakai, Tamon Kato, Tatsuya Saigusa, Ayako Okada, Hirohiko Motoki, Koichiro Kuwahara

**Affiliations:** 1 Department of Cardiovascular Medicine, Shinshu University School of Medicine, 3-1-1 Asahi, Matsumoto, Nagano 390-8621 Japan; 2 Department of Cardiology, Nagano Municipal Hospital, Nagano, Japan

**Keywords:** Coronary artery disease, Nutritional index, Prognosis

## Abstract

Nutritional status is a novel approach to prognostic assessment in patients with cardiovascular disease. However, assessment of nutritional status in elderly patients is challenging due to the significant differences between young patients. The TCBI (Triglycerides × Total cholesterol × Body Weight Index) is a novel and simple nutritional index for predicting long-term outcomes in patients with coronary artery disease. This retrospective study evaluated the efficacy of TCBI in 597 elderly (≥ 75 years) patients enrolled in the SHINANO 5 year registry. The SHINANO 5 year registry, a prospective observational multicenter cohort study, had enrolled 1501 consecutive patients who underwent elective/urgent percutaneous coronary intervention (PCI). In this study, patients were categorized into TCBI quartile groups. The primary endpoints were the occurrence of major adverse cardiac and cerebrovascular events (MACCE), including all-cause death, stroke, and myocardial infarction at 5 year. The mean duration of follow up was 4.3 ± 1.7 years. The average patient age was 80.9 ± 4.3 years. MACCE was observed in 61 (40.9%) patients in the lowest TCBI quartile group. Kaplan–Meier analysis demonstrated an inverse relationship between MACCE and TCBI (log-lank *P* < 0.001). Multivariate analysis demonstrated that low TCBI significantly predicted the incidence of MACCE (hazard ratio: 1.44, 95% confidence interval: 1.03–2.00; *P* = 0.031). The TCBI is useful in predicting long-term outcomes in elderly patients undergoing PCI.

**Electronic supplementary material:**

The online version of this article (10.1007/s00380-020-01659-0) contains supplementary material, which is available to authorized users.

## Introduction

Malnutrition has been identified as a significant predictor of mortality in various patient populations [1]. Furthermore, it is also associated with poor clinical outcomes of cardiovascular diseases, including stable coronary disease [2], chronic heart failure [3], end-stage renal disease [4], and peripheral artery disease [5]. Previous studies have demonstrated that several nutritional indicators, including serum albumin, body mass index (BMI), and cholesterol, are associated with the risk of cardiovascular events [6–8]. Recent studies have utilized the Geriatric Nutritional Risk Index (GNRI) and the Controlling Nutritional Status (CONUT) as nutritional indicators. Although these indicators are well accepted, they are not commonly used in the clinic setting since they require complex calculations and scoring. Doi et al. proposed a novel and simple nutritional index, the TCBI, which is calculated as Triglycerides (TG) × Total Cholesterol (TC) × Body Weight (BW) / 1000 [9]. TG, TC, and BW are commonly measured objective parameters in patients with cardiovascular disease. While the TCBI is useful in evaluating the nutritional status of patients with coronary artery disease (CAD), its utility in elderly patients remains unclear. This retrospective study aimed to investigate the utility of TCBI in predicting cardiovascular events in elderly patients who underwent percutaneous coronary intervention (PCI).

## Materials and methods

The SHINANO 5 year registry is a prospective, multi-center observational registry, which was designed to provide up to 5 years of clinical follow-up. This study enrolled 1665 consecutive patients who underwent PCI for CAD (including stable angina, ST-segment elevation myocardial infarction, non-ST-segment elevation myocardial infarction, and unstable angina) between August 2012 and July 2013 at 16 institutions in Nagano prefecture, Japan. The institutional review board approved the protocol, which was registered at the University Hospital Medical Information Network (UMIN000010070), and informed consent was obtained from all patients before enrollment. The study was performed in accordance with the Declaration of Helsinki.

**Fig. 1:**
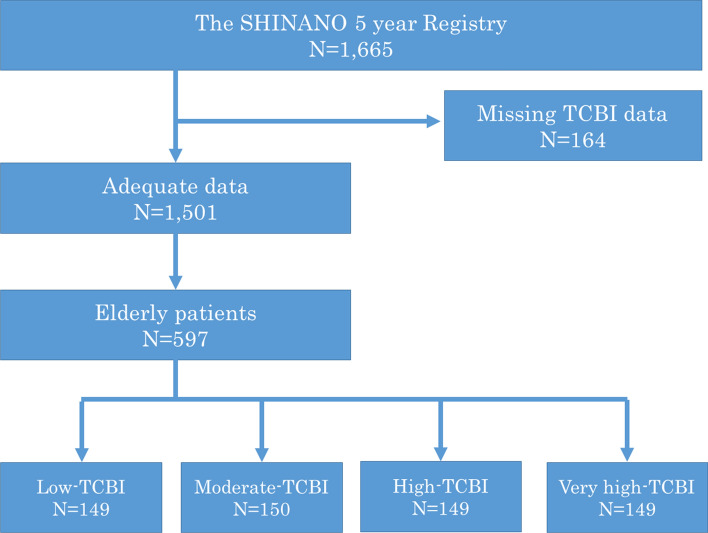
Study design

This study was an all-comer registry with no exclusion criteria. Among 1,665 patients registered in the SHINANO 5 year registry, we enrolled 1501; 164 were excluded owing to missing data concerning TCBI in this sub-analysis. Among these 1501 patients, 597 were elderly (Fig. 1)**.**

The primary endpoints were major adverse cardiac and cerebrovascular events (MACCE), including all-cause death, stroke, and myocardial infarction, in the follow-up period. In patients who had several events during follow-up, the time until the first event was considered during analysis.

The TCBI was calculated using the following formula:

TCBI = serum triglycerides (TG) (mg/dL) × serum total cholesterol (TC) (mg/dL) × body weight (BW) (kg) / 1000. The TG, TC, and BW were measured on admission to hospital. The 597 elderly patients were categorized into 4 groups as follows: low-TCBI (TCBI < 590.5; *n* = 149), moderate-TCBI (590.5 ≤ TCBI < 928; *n* = 150), high-TCBI (928 ≤ TCBI < 1451; *n* = 149), and very high-TCBI (1451 ≤ TCBI; *n* = 149) groups.

Continuous variables are expressed as the mean ± standard deviation when normally distributed, and as the median and interquartile range when non-normally distributed. Categorical variables are expressed as numbers with percentages. These were compared using the Kruskal–Wallis and chi-square tests, respectively. Kaplan–Meier curves were calculated from the date of PCI to the occurrence of MACCE, and were compared using the log-rank test. A Cox proportional hazards regression analysis was performed to identify predictors of MACCE among clinical characteristics and risk factors. Multivariable analysis was performed to adjust for the effects of baseline risk factors. We considered age; male gender; statin use; acute coronary syndrome (ACS); low left ventricular ejection fraction (LVEF; < 40%); and conventional coronary risk factors, such as chronic kidney disease (CKD), peripheral artery disease (PAD), diabetes mellitus (DM), and hypertension (HT), as candidates in our multivariable analysis. Since TCBI is a novel nutritional index, we also evaluated its usefulness in all 1,501 patients after classifying them according to TCBI quartile (Supplementary Fig. 1 and Table 1). All analyses were performed using SPSS version 25.0 (SPSS, Chicago, IL) software package.

## Results

**Fig. 2:**
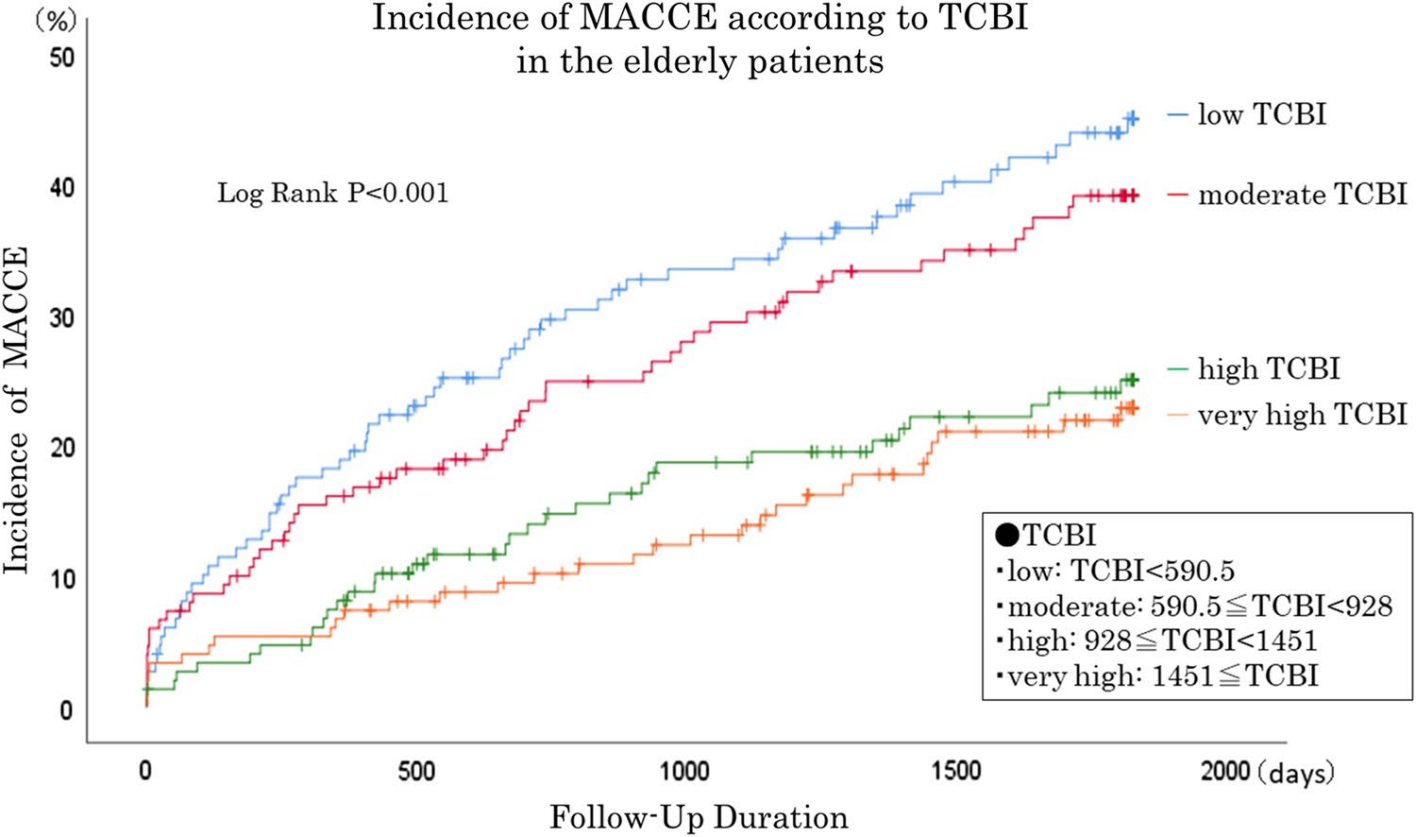
Kaplan–Meier curves for MACCE in elderly patients. *MACCE* major adverse cardiac and cerebrovascular events

**Fig. 3:**
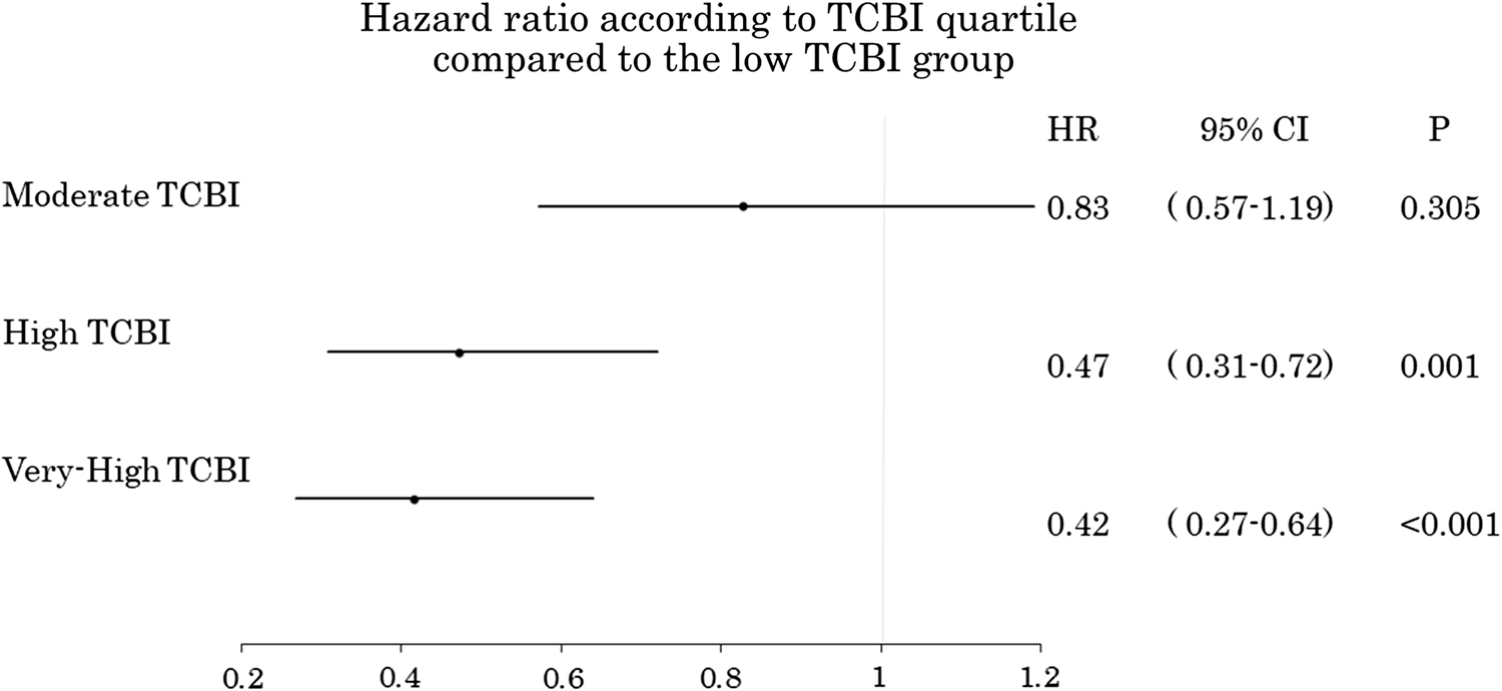
Hazard ratio according to TCBI quartile compared with the low TCBI group. *CI* confidence interval; *HR* hazard ratio

The patients’ baseline clinical characteristics are listed in Table 1. The average age was 80.9 ± 4.3 years, and 65.7% were male. Those in the lower TCBI quartiles were older with lower body weight compared to the other groups. In terms of coronary risk factors, hypertension and current smoking were did not differ among the 4 groups, while the rate of dyslipidemia was lower in the low TCBI group. The prevalence of DM showed no differences between the groups, while hemoglobin A1c tended to be lower in the low TCBI group. The low TCBI group also included several ACS patients and the LVEF was also lower. Figure 2 shows the Kaplan–Meier analysis. The low-TCBI group (< 590.5, *n* = 149) showed a poorer prognosis than the other groups (MACCE incidence vs. the moderate, high, and very high TCBI groups: 40.9% vs. 36.0% vs. 22.1% vs. 20.8%, respectively; *P* < 0.001). Figure 3 shows the hazard ratio [HR] according to TCBI quartile. The HR in the moderate versus low TCBI group was 0.83, but the difference was not significant [95% confidence interval (CI) 0.57–1.19, *P* = 0.305]. Further elevation of TCBI was associated with a significant decrease in HR; the high TCBI group showed a HR of 0.47 (95% CI 0.31–0.72, *P* = 0.001), while the very high group had an HR of 0.42 (95% CI 0.27–0.64, *P* < 0.001). The results of the univariate and multivariate analyses are shown in Table 2. The univariate Cox proportional hazards analysis showed that low TCBI, age, statin use, ACS, low LVEF, and CKD were associated with a higher risk of MACCE. The multivariate analysis identified low TCBI as an independent predictor of poor prognosis (HR: 1.44; 95% CI 1.03–2.01; *P* = 0.031). Additional independent predictors of MACCE included age (HR: 1.06; 95%CI: 1.03–1.10; *P* = 0.001), statin use (HR: 0.46; 95% CI 0.32–0.67; *P* < 0.001), and low LVEF (HR: 2.05; 95% CI 1.40–3.01; *P* < 0.001). We subsequently analyzed the entire 1,501 patient cohort after dividing them by TCBI quartile (Supplementary Fig. 1 and Table 1). On Kaplan–Meier analysis, the low-TCBI group demonstrated a poorer prognosis than other groups (Supplementary Fig. 2). Multivariate Cox proportional analyses confirmed that low TCBI was independently associated with poor prognosis (HR: 1.36; 95% CI 1.06–1.74; *P* = 0.015) (Supplementary Table 2).

**Table 1:** Baseline characteristics

	Low-TCBI (*N* = 149)	Moderate-TCBI (*N* = 150)	High-TCBI (*N* = 149)	Very-High (*N* = 149)	*P* value
Age	82.0 (79, 86)	81.0 (77, 84)	80.0 (77, 83)	79.0 (77, 82)	< 0.001
Gender, male	96, 64.4	101, 67.3%	97, 65.1%	98, 65.8%	0.959
Body weight, kg	49.2 (43.0, 55.5)	55.2 (49.9, 61.3)	57.0 (50.3, 63.8)	61.6 (54.4, 68.2)	< 0.001
Body mass index, kg/m^2^	20.7 (18.6, 22.7)	22.5 (20.7, 24.2)	23.2 (21.5, 25.1)	24.9 (22.7, 26.9)	< 0.001
Hypertension	112, 75.2%	123, 82.0%	121, 81.2%	119, 79.9%	0.462
Systolic blood pressure, mmHg	125.0 (110.2, 138.0)	125.5 (113.8, 142.3)	129.0 (117.0, 143.0)	130.0 (120.0, 143.0)	0.03
Diabetes mellitus	45, 30.2%	45, 30.0%	50, 33.6%	53, 35.6%	0.683
HbA1c, %	5.7 (5.4, 6.2)	5.9 (5.5, 6.2)	6.0 (5.6, 6.5)	6.0 (5.7, 6.6)	< 0.001
Dyslipidemia	61, 40.9%	70, 46.7%	91, 61.1%	97, 65.5%	< 0.001
Triglycerides, mg/dl	57.0 (46.0, 70.0)	82.0 (75.8, 93.5)	115.0 (100.5, 131.0)	183.0 (144.5, 211.5)	< 0.001
Total cholesterol, mg/dl	146.0 (126.0, 165.5)	162.0 (143.0, 183.3)	180.0 (153.0, 201.0)	198.0 (175.5, 226.0)	< 0.001
Peripheral artery disease	27, 18.1%	18, 12.1%	25, 16.8%	21, 14.1%	0.47
Current smoking	9, 6.1%	8, 5.4%	14, 9.5%	15, 10.3%	0.306
Acute coronary syndrome	84, 56.8%	63, 42.0%	59, 39.6%	38, 25.5%	< 0.001
Multi-vessel disease	62, 41.6%	55, 36.7%	68, 45.6%	55, 36.9%	0.333
Number of diseases vessels	1.0 (1.0, 2.0)	1.0 (1.0, 2.0)	1.0 (1.0, 2.0)	1.0 (1.0, 2.0)	0.49
LVEF, %	58.0 (47.0, 66.0)	61.2 (48.9, 70.3)	62.0 (55.0, 69.0)	65.0 (58.1, 73.0)	< 0.001
LVEF < 40%	23, 16.1%	25, 17.2%	14, 9.7%	10, 7.0%	0.023
Chronic kidney disease	84, 56.4%	82, 54.7%	86, 57.7%	95, 63.8	0.411
Cre, mg/dl	0.9 (0.7, 1.3)	0.9 (0.7, 1.1)	0.9 (0.7, 1.1)	0.9 (0.8, 1.1)	0.879
eGFR, ml/min/1.73*2	55.1 (38.5, 71.1)	58.7 (41.1, 69.7)	57.1 (44.6, 67.8)	55.2 (45.3, 64.0)	0.813
CRP, mg/dl	0.2 (0.1, 0.6)	0.1 (0.1, 0.6)	0.1 (0.1, 0.3)	0.1 (0.1, 0.4)	0.381
Medication					
Beta blocker	27, 18.1%	36, 24.0%	42, 28.2%	45, 30.2%	0.08
ACE-inhibitor / ARB	84, 56.8%	63, 42.0%	59, 39.6%	38, 25.5%	< 0.001
Statin	55, 36.9%	50, 33.3%	69, 46.3%	63, 42.3%	0.104
EPA	5, 3.4%	3, 2.0%	5, 3.4%	3, 2.0%	0.791
Target lesion of PCI					
LMT	5, 3.4%	3, 2.0%	5, 3.4%	2, 1.4%	0.434
LAD	63, 42.6%	66, 44.9%	52, 35.6%	74, 50.0%	
LCx	19, 12.8%	26, 17.7%	26, 17.8%	21, 14.2%	
RCA	61, 41.2%	52, 35.4%	62, 42.5%	51, 34.5%	
Bypass graft	0, 0%	0, 0%	1, 0.7%	0, 0%	
Total stent length	20.0 (16.0, 30.0)	24.0 (16.0, 32.0)	22.5 (15.0, 32.0)	23.0 (16.0, 30.8)	0.644

*CRP* C-reactive protein, *EPA* eicosapentaenoic acid, *LAD* left anterior descending artery, *LCx* left circumflex artery, *LMT* left main coronary trunk, *LVEF* left ventricular ejection fraction, *PCI* percutaneous coronary intervention, *RCA* right coronary artery

**Table 2:** Cox proportional hazards analysis of MACCE

	Unadjusted HR (95% CI)	*P*	Adjusted HR (95% CI)	*P*
Low TCBI	1.780 (1.306–2.425)	< 0.001	1.440 (1.034–2.006)	0.031
Age	1.097 (1.062–1.133)	< 0.001	1.063 (1.025–1.101)	0.001
Gender, male	0.920 (0.677–1.250)	0.592	0.940 (0.676–1.308)	0.714
Statin	0.463 (0.333–0.645)	< 0.001	0.463 (0.322–0.667)	< 0.001
Acute coronary syndrome	1.649 (1.229–2.212)	0.001	1.184 (0.848–1.653)	0.321
LVEF < 40%	2.206 (1.533–3.173)	< 0.001	2.050 (1.396–3.010)	< 0.001
Chronic kidney disease	1.481 (1.087–2.018)	0.013	1.247 (0.901–1.727)	0.183
Peripheral artery disease	1.361 (0.937–1.978)	0.106	1.212 (0.804–1.828)	0.358
Diabetes mellitus	1.240 (0.916–1.680)	0.164	1.211 (0.878–1.669)	0.244
Hypertension	1.019 (0.704–1.474)	0.920	1.045 (0.707–1.546)	0.824

*LVEF* left ventricular ejection fraction, *MACCE* major adverse cardiac and cerebrovascular events

## Discussion

In this study, we evaluated the utility of TCBI for predicting cardiovascular events in elderly patients with CAD. The findings suggest a definite association between TCBI levels and the incidence of MACCE. While previous studies have shown a correlation between TCBI levels and different causes of mortality, this is the first report to show the relationship between MACCE and TCBI. It also suggests that the TCBI can be useful in predicting long-term outcomes of elderly patients who undergo PCI for CAD. Although the focus remains on the management of conventional risk factors, including DM and dyslipidemia, nutritional index assessment is gaining popularity as a residual risk factor. The GNRI, CONUT, Prognostic Nutritional Index (PNI), Malnutrition Inflammation Score (MIS), and Mini Nutritional Assessment (MNA) are known as nutritional indices. In particular, the GNRI, which considers albumin levels, is frequently employed as a nutritional index in patients with chronic heart failure [3, 10, 11]. In recent years, the GNRI has also been used in patients with CAD [2, 12, 13]. The TCBI is a novel nutritional index based on BW, TC, and TG, all of which are risk factors for arteriosclerosis. A previous study found a correlation between TCBI and GNRI and observed an association between low TCBI and high all-cause, cardiovascular, and cancer-related mortality. Therefore, TCBI is considered to be a useful prognostic indicator in CAD patients [9]. Moreover, it may also be a valuable indicator in patients with a critical and hemodynamically unstable cardiovascular disease requiring percutaneously implantable mechanical circulatory support devices [14]. Unlike the GNRI, the TCBI comprises general examination variables relevant to patients with CAD. Therefore, it can be used to determine prognosis from a nutritional perspective using variables that are commonly used for risk management.

The nutritional status differs between younger and older individuals. Serum albumin levels are a well-characterized marker of nutritional status, but they decrease with age [15, 16]. This emphasizes how the approach to nutritional status assessment should differ for elderly and young patients. In this study, we focused on the utility of TCBI in CAD patients at least 75 years of age. Our most significant finding was that low TCBI is useful in predicting the long-term incidence of MACCE. Furthermore, the prevalence of dyslipidemia, TG, TC, and BW was significantly lower in the low TCBI group. Low lipid levels and BW, as markers of nutritional status, are associated with a poor prognosis. The optimal approach to lipid management in elderly patients remains debatable. Although the 2017 Japan Atherosclerosis Society Guidelines for Prevention of Atherosclerotic Cardiovascular Disease advocate strict lipid management as has been historically performed, it also proposes that patients ≥ 75 years should receive a more tailored approach. This may be because the association between lipid management and CAD is controversial [17–20], and the significance of the primary prevention effect has not been fully understood in the elderly. It should be noted that this study focused on secondary prevention in patients with CAD and that the results were paradoxical in that the group with the poorest prognosis had low levels of TC and TG. This indicates that, in elderly persons, alternative lipid management strategies that focus on nutritional status rather than conventional lipid-lowering therapy may be more appropriate. The BW findings also showed similar trends. The concept of the obesity paradox suggests that overweight or obese patients with heart failure [21, 22], CAD [23, 24], and CKD [25] have a better prognosis. Paradoxical relationships between clinical outcomes and both lipid parameters and BW have also been observed in cardiovascular patients. More recently, sarcopenia and frailty have received attention as prognostic factors, and their definitions include muscle and weight loss. In this study, the low TCBI group also had lower BW than the other groups, which may suggest a relationship between TCBI and frailty. In view of these reports, it remains unclear whether increases in cholesterol levels and BW may be associated with a more positive prognosis. Nevertheless, TCBI may help predict the risk of MACCE in patients with CAD.

## Study limitations

This study has several limitations. First, the study population was small and comprised a single ethnic group. Second, lipid-lowering therapy, such as statins and ezetimibe, affect the TCBI value; therefore, further analyses that consider the effect of these medications are required. Third, data on TCBI changes during follow-up were lacking. Since these changes may influence the clinical outcome, their evaluation is important. Fourth, high levels of TC, TG, and BW are regarded as risk factors for arteriosclerosis. Considering the previously mentioned paradoxical relationships, the association between TCBI and arteriosclerosis requires further investigation. Fifth, this study did not evaluate the patients’ degree of frailty, which should be investigated in further studies to determine if they are associated with TCBI.

## Conclusions

TCBI is a useful index for predicting the long-term outcomes of elderly patients with CAD who undergo PCI. Further studies with more diverse cohorts are needed to validate our findings.

## Electronic supplementary material

Below is the link to the electronic supplementary material.

Supplementary file1 Supplementary Figure 1: Study design of the overall cohort analysis (TIF 14450 kb)

Supplementary file2 Supplementary Figure 2: Kaplan-Meier curves for MACCE in all patients. MACCE: Major Adverse Cardiac and Cerebrovascular Events (TIF 14777 kb)

Supplementary file3 (DOCX 24 kb)

Supplementary file4 (DOCX 17 kb)
