# Supplementary figures and images for: Impact of nutritional index on long-term outcomes of elderly patients with coronary artery disease: sub-analysis of the SHINANO 5 year registry
Source: Heart Vessels. 2020 Jun 30;36(1):7–13. doi: 10.1007/s00380-020-01659-0 (PMC7788017; doi:10.1007/s00380-020-01659-0)

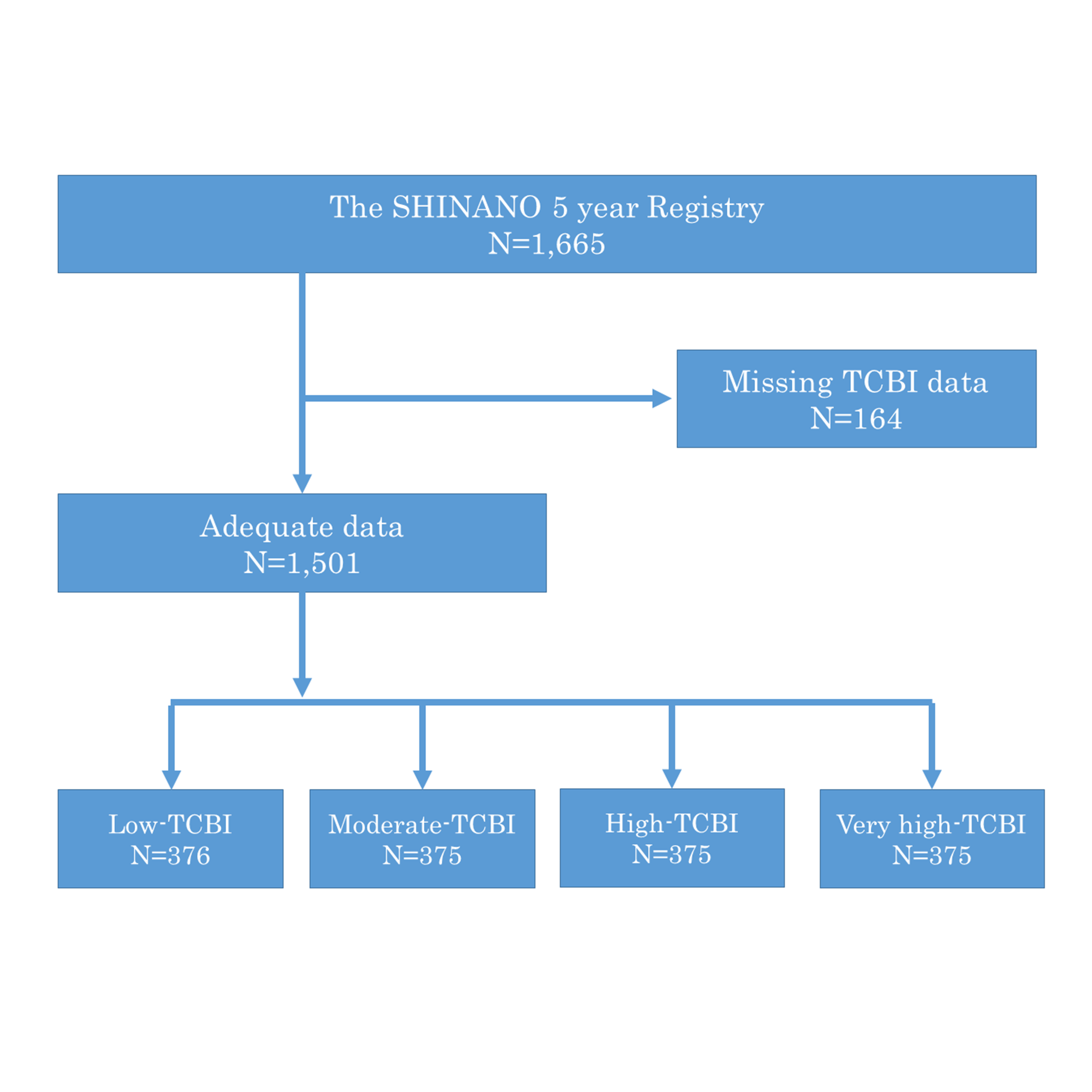

Supplement: Supplementary file 1 — Supplementary file1 Supplementary Figure 1: Study design of the overall cohort analysis (TIF 14450 kb) [file 380_2020_1659_MOESM1_ESM.tif]

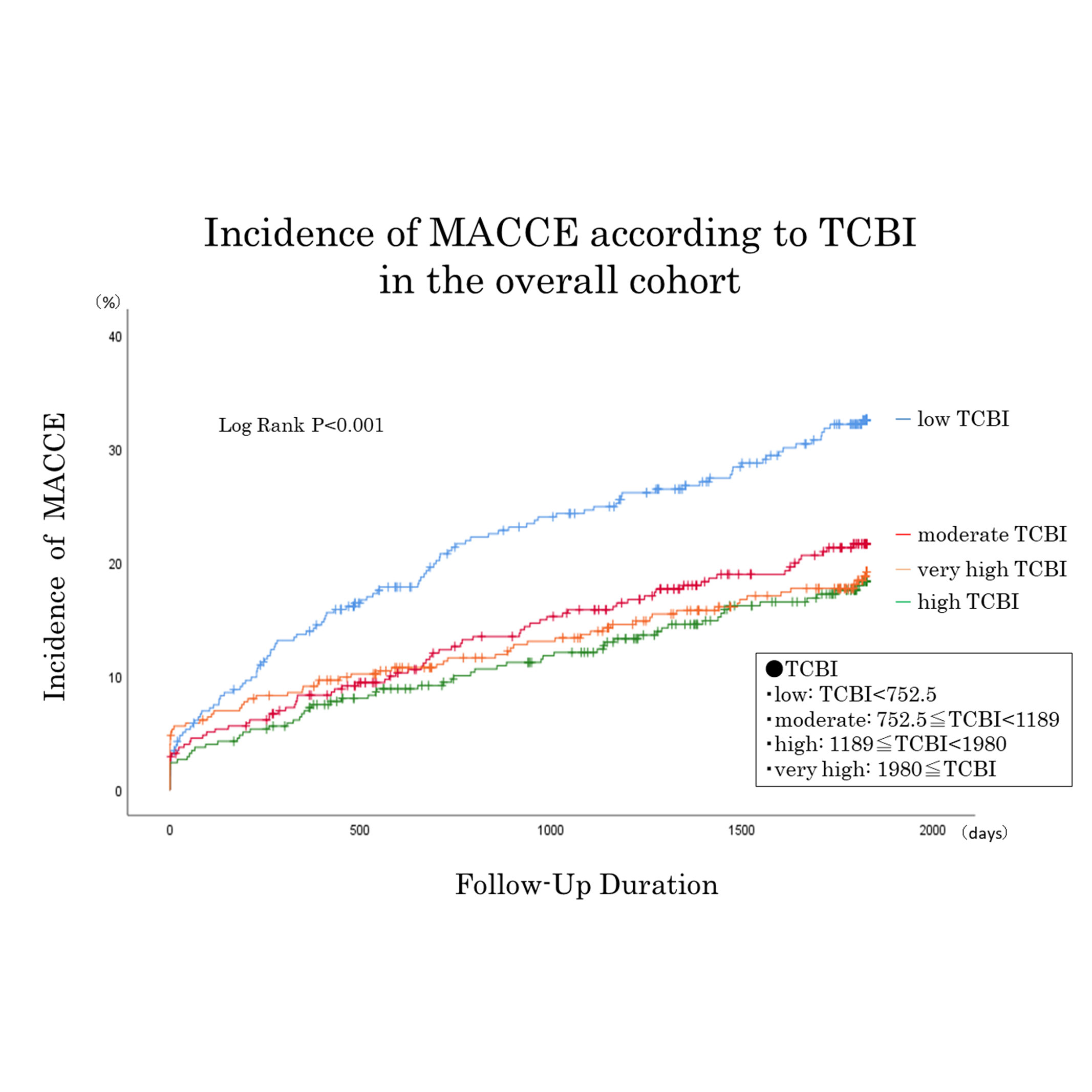

Supplement: Supplementary file 2 — Supplementary file2 Supplementary Figure 2: Kaplan-Meier curves for MACCE in all patients. MACCE: Major Adverse Cardiac and Cerebrovascular Events (TIF 14777 kb) [file 380_2020_1659_MOESM2_ESM.tif]
