# Supplementary material for: Impact of nutritional index on long-term outcomes of elderly patients with coronary artery disease: sub-analysis of the SHINANO 5 year registry
Source: Heart Vessels. 2020 Jun 30;36(1):7–13. doi: 10.1007/s00380-020-01659-0 (PMC7788017; doi:10.1007/s00380-020-01659-0)
Supplement: Supplementary file 3 — Supplementary file3 (DOCX 24 kb) [file 380_2020_1659_MOESM3_ESM.docx]

**Supplementary Table 1. Baseline characteristics of the overall cohort**

|  | **Low-TCBI**  **(N=376)** | **Moderate-TCBI**  **(N=375)** | **High-TCBI**  **(N=375)** | **Very High-TCBI**  **(N=375)** | **P-value** |
| --- | --- | --- | --- | --- | --- |
| Age | 77.0 (70, 83) | 73.0 (66, 80) | 71.0 (63, 77) | 65.0 (58, 72) | <0.001 |
| Gender, male | 267, 71.0% | 284, 75.7% | 287, 76.5% | 310, 82.7% | 0.002 |
| Body weight, kg | 52.9 (47.1, 60.0) | 59.6 (53.4, 66.6) | 63.5 (56.3, 70.0) | 69.0 (62.3, 75.6) | <0.001 |
| Body mass index, kg/m^2^ | 21.3 (19.4, 23.2) | 23.3 (21.7, 25.0) | 24.2 (22.2, 26.5) | 25.3 (23.5, 27.6) | <0.001 |
| Hypertension | 274, 72.9% | 286, 76.3% | 290, 77.3% | 275, 73.3% | 0.413 |
| Systolic blood pressure, mmHg | 122.0 (109.0, 138.8) | 129.0 (114.0, 143.0) | 127.0 (116.0, 139.0) | 128.0 (116.0, 141.0) | <0.001 |
| Diabetes mellitus | 127, 33.8% | 137, 36.5% | 129, 34.4% | 163, 43.5% | 0.023 |
| HbA1c, % | 5.8 (5.4, 6.4) | 5.9 (5.6, 6.5) | 5.9 (5.6, 6.5) | 6.2 (5.7, 7.1) | <0.001 |
| Dyslipidemia | 171, 45.5% | 218, 58.1 | 228, 60.8% | 295,78.9% | <0.001 |
| Triglyceride, mg/dl | 66.0 (51.0, 79.0) | 97.0 (83.0, 114.0) | 131.0 (113.0, 155.0) | 208.0 (172.0, 258.0) | <0.001 |
| Total cholesterol, mg/dl | 150.0 (131.0, 169.9) | 164.0 (145.0, 187.0) | 182.0 (163.0, 203.0) | 208.0 (184.0, 234.0) | <0.001 |
| Peripheral artery disease | 64, 17.0% | 40, 10.7% | 31, 8.3% | 30, 8.0% | <0.001 |
| Current smoking | 47, 12.7% | 51, 13.7% | 61, 16.5% | 112, 30.2% | <0.001 |
| Acute coronary syndrome | 186, 49.6% | 133, 35.5% | 145, 38.7% | 163, 43.55 | <0.001 |
| Multi-vessel disease | 139, 37.1% | 149, 39.7% | 145, 38.7% | 142, 37.9% | 0.892 |
| Number of diseased vessels | 1.0 (1, 2) | 1.0 (1, 2) | 1.0 (1, 2) | 1.0 (1, 2) | 0.901 |
| LVEF, % | 59.6 (50.0, 67.3) | 63.0 (52.9, 70.0) | 64.0 (54.6, 70.3) | 63.0 (51.6, 68.0) | <0.001 |
| LVEF<40% | 56, 15.3% | 49, 13.6 | 37, 10.1% | 39, 10.8% | 0.111 |
| Chronic kidney disease | 181, 48.1% | 185, 49.3% | 167, 44.5% | 150, 40.0% | 0.046 |
| Cre, mg/dl | 0.9 (0.7, 1.2) | 0.9 (0.8, 1.1) | 0.9 (0.8, 1.1) | 0.9 (0.7, 1.1) | 0.594 |
| eGFR, ml/min/1.73*2 | 60.9 (41.8, 74.0) | 60.1 (46.1, 73.0) | 62.3 (50.1, 72.9) | 64.6 (54.1, 78.0) | 0.001 |
| CRP, mg/dl | 0.1 (0.0, 0.5) | 0.1 (0.0, 0.4) | 0.1 (0.0, 0.3) | 0.1 (0.0, 0.3) | 0.752 |
| **Medications** |  |  |  |  |  |
| Beta blocker | 85, 22.6% | 109, 29.1% | 91, 24.3% | 97, 25.9% | 0.212 |
| ACE-inhibitor / ARB | 181, 48.1% | 188, 50.1% | 197, 52.5% | 186, 49.6% | 0.681 |
| Statin | 146, 38.8% | 187, 49.9% | 171, 45.6% | 159, 42.4% | 0.018 |
| EPA | 10, 2.7% | 13, 3.5% | 15, 4.0% | 12, 3.2% | 0.779 |
| **Target lesion of PCI** |  |  |  |  |  |
| LMT | 8, 2.1% | 10, 2.7% | 7, 1.9% | 6, 1.6% | 0.305 |
| LAD | 153, 41.0% | 157, 42.4% | 188, 50.5% | 171, 45.7% |  |
| LCx | 59, 15.8% | 67, 18.1% | 54, 14.55 | 60, 16.0% |  |
| RCA | 151, 40.5% | 136, 36.8% | 120, 32.3% | 136, 36.4% |  |
| Bypass graft | 2, 0.5% | 0, 0% | 3, 0.8% | 1, 0.1% |  |
| Total stent length | 22.0 (16.0, 30.0) | 22.0 (16.0, 32.0) | 22.0 (16.0, 30.0) | 23.0 (16.0, 30.0) | 0.856 |

CRP: C-reactive protein, EPA: eicosapentaenoic acid, LAD: left anterior descending artery, LCx: left circumflex artery, LMT: left main coronary trunk, LVEF: left ventricular ejection fraction, PCI: percutaneous coronary intervention, RCA: right coronary artery.
