# Supplementary material for: Impact of nutritional index on long-term outcomes of elderly patients with coronary artery disease: sub-analysis of the SHINANO 5 year registry
Source: Heart Vessels. 2020 Jun 30;36(1):7–13. doi: 10.1007/s00380-020-01659-0 (PMC7788017; doi:10.1007/s00380-020-01659-0)
Supplement: Supplementary file 4 — Supplementary file4 (DOCX 17 kb) [file 380_2020_1659_MOESM4_ESM.docx]

**Supplementary Table 2. Cox proportional hazards analysis of MACCE in the overall cohort**

|  | **unadjusted HR (95% CI)** | **P** | **adjusted HR (95% CI)** | **P** |
| --- | --- | --- | --- | --- |
| Low TCBI | 1.798 (1.429-2.263) | <0.001 | 1.359 (1.062-1.740) | 0.015 |
| Age | 1.044 (1.032-1.056) | <0.001 | 1.032 (1.019-1.045) | <0.001 |
| Gender, male | 0.865 (0.673-1.112) | 0.257 | 1.036 (0.794-1.353) | 0.794 |
| Statin | 0.477 (0.375-0.607) | <0.001 | 0.487 (0.375-0.632) | <0.001 |
| Acute coronary syndrome | 1.482 (1.189-1.847) | <0.001 | 1.305 (1.024-1.662) | 0.031 |
| LVEF<40% | 2.542 (1.955-3.307) | <0.001 | 2.247 (1.701-2.969) | <0.001 |
| Chronic kidney disease | 2.230 (1.776-2.802) | <0.001 | 1.746 (1.366-2.232) | <0.001 |
| Peripheral artery disease | 1.719 (1.282-2.304) | <0.001 | 1.213 (0.883-1.665) | 0.233 |
| Diabetes mellitus | 1.366 (1.095-1.705) | 0.006 | 1.351 (1.069-1.708) | 0.012 |
| Hypertension | 1.208 (0.925-1.577) | 0.164 | 1.074 (0.809-1.425) | 0.623 |

LVEF: left ventricular ejection fraction, MACCE: major adverse cardiac and cerebrovascular events
